# Supplementary material for: A Natural Quinazoline Derivative from Marine Sponge Hyrtios erectus Induces Apoptosis of Breast Cancer Cells via ROS Production and Intrinsic or Extrinsic Apoptosis Pathways
Source: Mar Drugs. 2019 Nov 23;17(12):658. doi: 10.3390/md17120658 (PMC6950652; doi:10.3390/md17120658)
Supplement: Supplementary file 1 [file marinedrugs-17-00658-s001.pdf]

Supplementary File

A natural quinazoline derivative from marine sponge *Hyrtios erectus* induces apoptosis of breast cancer cells via ROS production and intrinsic or extrinsic apoptosis pathways

Arun Kumar De[1,2,*], Ramachandran Muthiyan[2], Samiran Mondal[3], Nilkamal Mahanta[4,5], Debasis Bhattacharya[1], Perumal Ponraj[1], K. Muniswamy[1], Anandamoy Kundu[1], Madhu Sudhan Kundu[1], Jai Sunder[1], D. Karunakaran[6], Asit Kumar Bera[7], Sibnarayan Dam Roy[8] and Dhruba Malakar[9,*]

Effect of the compound A on proliferation of MDA-MB-231 and MDA-MB-415 cell lines in vitro

An MTT assay was performed to assess the anti-proliferative effect of the compound on two adenocarcinoma cell lines. Exponentially growing cells were exposed to various concentrations of the compound for 24 h and 48 h. The results showed that the compound inhibited the proliferation of the both the cell lines in a concentration dependent manner (Supplementary Figure S1 A-D). The determined half maximal inhibitory concentration values (IC_{50}) of the compound on the two cell lines is presented in Supplementary Table S1.

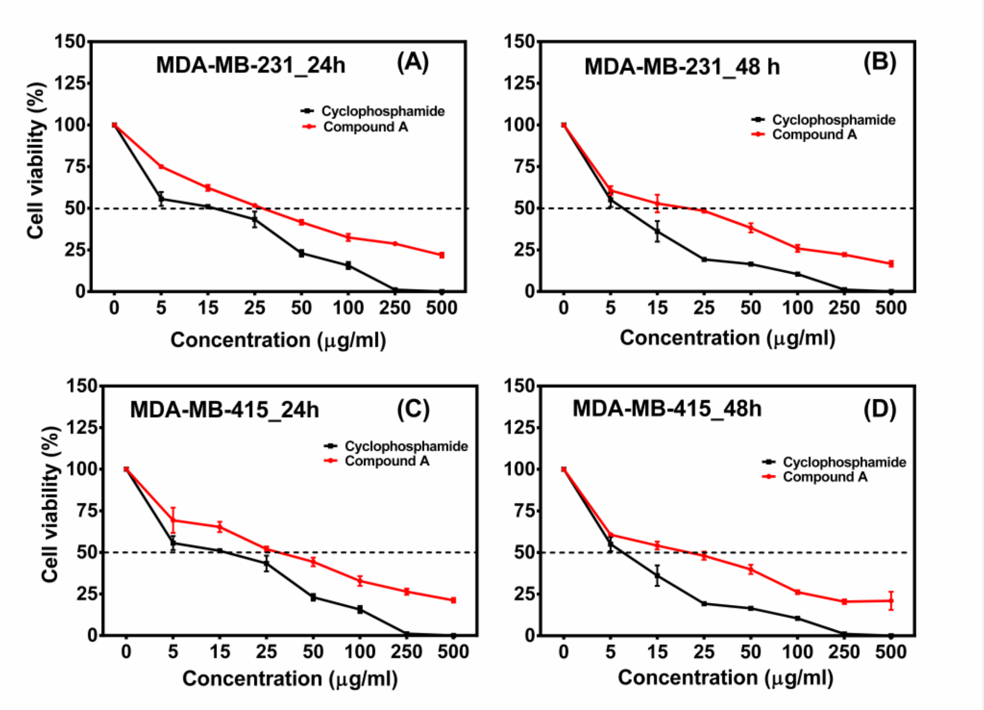

Supplementary Figure S1: Cytotoxic effect of the quinazoline derivative (Compound A) on MDA-MB-231 and MDA-MB-415 cell lines. An MTT assay was done to evaluate the cytotoxic effect of the compound on MDA-MB-231 and MDA-MB-415. Cyclophosphamide was used as a standard anti-cancer drug. (A) Effect of compound A and Cyclophosphamide on viability of MDA-MB-231 cells (24 h), (B) Effect of compound A and Cyclophosphamide on viability of MDA-MB-231 cells (48 h), (C) Effect of compound A and Cyclophosphamide on viability of MDA-MB-415 cells (24 h), (D) Effect of compound A and Cyclophosphamide on viability of MDA-MB-415 cells (48 h)

Supplementary Table S1 IC50 values of the quinazoline derivative (Compound A) compound against MDA-MB-231 and MDA-MB-415 cell lines.

Compound	Cell Line	Cell Type	IC50 (μg/ml)	
			24 h	48 h
Compound A	MDA-MB-231	Adenocarcinoma, mammary glad	25.45±1.67	18.17±1.21
Compound A	MDA-MB-415	Adenocarcinoma, mammary glad	28.76±1.83	22.34±1.54

Data are presented as mean ± SD of three independent experiments.

Apoptotic potential of the compound on MDA-MB-231 and MDA-MB-415 cell lines

The apoptotic potential of the compound on MDA-MB-231 and MDA-MB-415 cell lines was evaluated by flow cytometry. The results indicated that the compound induced apoptosis of the two cell lines in a concentration dependant manner. Significant increase in apoptotic cell percentage was observed in MDA-MB-231 cells treated with 15 μg/ml of the compound onwards as compared to those of control. In case of MDA-MB-415 cells, a little more concentration of the compound (25 μg/ml) was required for significant increase of apoptotic cells as compared to control (Supplementary Figure S2).

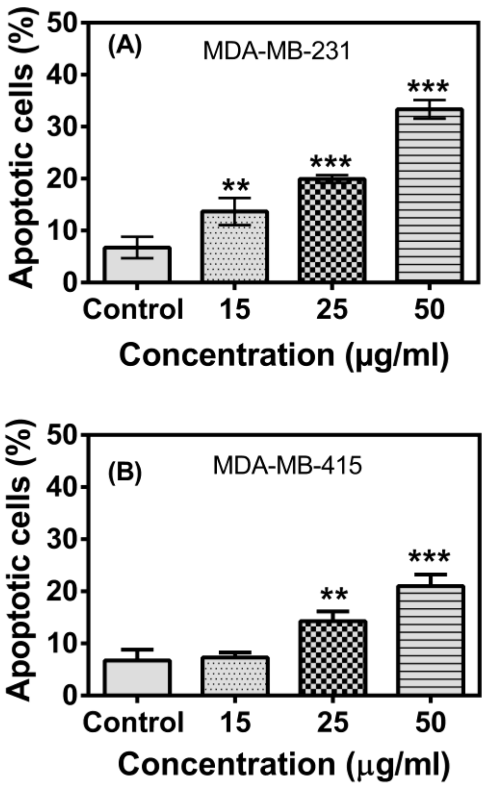

Supplementary Figure S2: Apoptotic potential of the compound against MDA-MB-231 and MDA-MB-415 cell lines. Apoptotic cells were stained by Annexin-V and detected by Flow Cytometry; The histogram shows the mean Annexin V-positive cells (mean ± SD) of three experiments. One-way analysis of variance (ANOVA)

followed by Dunnett post-test was performed to find out significant difference among control and treatments. * denotes $p \leq 0.05$; ** denotes $p \leq 0.01$; *** denotes $p < 0.001$

Apoptosis on MCF-10A treated with compound A

The effect of the compound A on apoptosis of the MCF-10A cells was investigated by Flow Cytometry and no significant difference of apoptotic cell percentage between treated cells and control was found (Supplementary Figure S3). This indicates that Compound A was less cytotoxic towards MCF-10A cells. MCF-10A cells cultured in absence of the compound A showed 4.74 % apoptotic cells and 5.33, 6.61 and 7.01 % following the addition of 15, 25 and 50 μg/ml compound A for 24 h treatment .

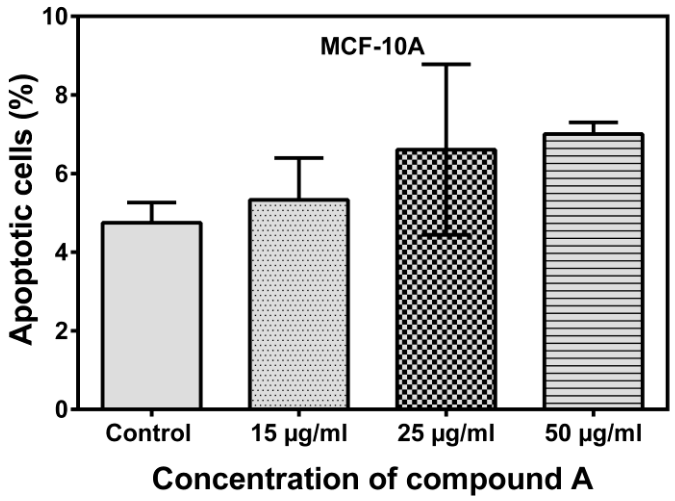

Supplementary Figure S3. Apoptotic potential of the Compound A on MCF-10A cells. The histogram shows the mean Annexin V-positive MCF-10A cells (mean ± SD) of three experiments.

ROS production on MCF-10A treated with compound A

Effect of Compound A on ROS production in MCF-10A cells was evaluated and no significant differences in ROS production between treated cells and control cell was observed (Supplementary Figure S4)

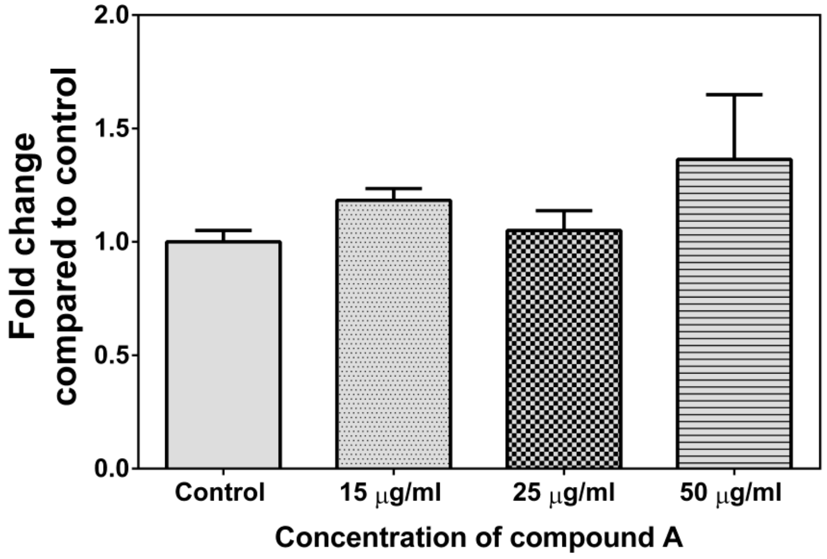

Supplementary Figure S4. Effect of compound A on generation of ROS in MCF-10A cells. Data are shown as mean ± SD of three independent experiments.

Effects of cyclophosphamide on the expression of apoptotic-related genes in MCF-7 cells

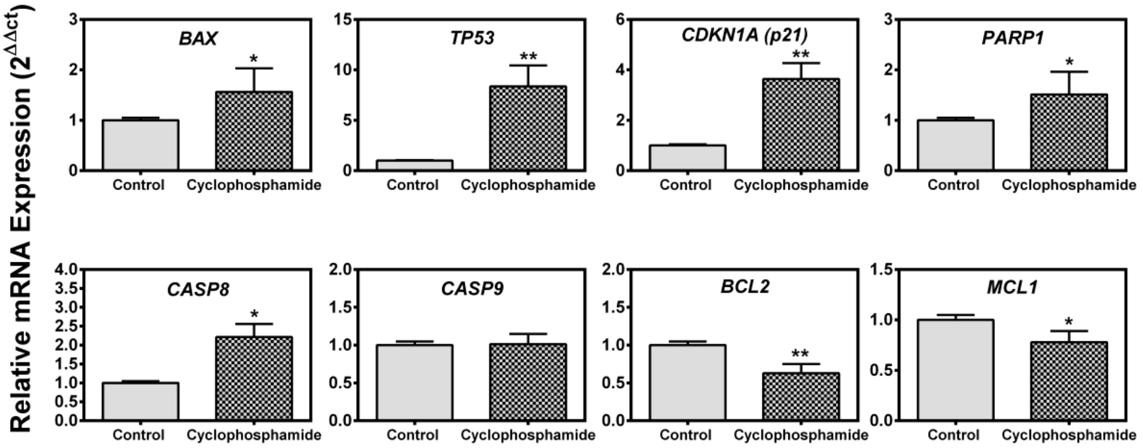

Supplementary Figure S5 Effect of the cyclophosphamide on the expression of apoptosis related genes in MCF-7 Cells. Real time RT-PCR was performed to analyze the expression of genes involved in apoptosis pathway. The values and error bars represent average and standard deviations (SD) of three independent sets of experiments. One-way analysis of variance (ANOVA) followed by Dunnett post-test was performed to find out significant difference among control and treatments. * denotes $p \leq 0.05$; ** denotes $p \leq 0.01$.

Supplementary Table S2 Information of the primers used for Real time PCR

Gene	Forward primer	Reverse primer	References
GAPDH	5' TGAACGGGAAG CTCACTGG 3'	5' GCTTCACCACCTTCTTGAT GTC 3'	[1]
Bax	5'TTTGCTTCAGGGTTTCATCCA 3′	5′ CTCCATGTTACTGTCCAGTTCGT 3′	[2]
Bcl-2	5′ CATGTGTGTGGAGAGCGTCAAC 3′	5′ CAGATAGGCACCCAGGGTGAT 3′	[2]
Caspase-3	5′ TATGGTTTTGTGATGTTTGTCC 3'	5′ TAGATCCAGGGGCATTGTAG 3′	[2]
Caspase-8	5′ CTACCAACTCATGGACCACAG 3′	5′ GTGACTGGATGTACCAGGTTC 3′	[2]
Caspase-9	5′ TACAGCTGTTCAGACTCTAGTA 3′	5′ AAATATGTCCTGGGGTAT 3′	[2]
P21	5'TTAGCAGCGGAACAAGGAGT3'	3'AAGCCGAGAGAAAACAGTCCA5'	[3]

PARP1	5'TATCGAGTCGAGTACGCCAA3'	3'AAACTACCTTTTCAGGGTGTG5'	[3]
Caspase-2	5' TGGGTGCGAGGAGAGTGATG 3'	5' TGGCGGCAGTCCCTTTGAG 3'	[4]
Caspase-7	5' GTCTCACCTATCCTGCCCTCAC 3'	5' TTCTTCTCCTGCCTCACTGTCC 3'	[4]
Bcl-xL	5'GTTCCCTTTCCTTCCATCC 3'	5' TAGCCAGTCCAGAGGTGAG 3'	[5]
BID	5'CCTTGCTCCGTGATGTCTTTC3'	5' TCCGTTCAGTCCATCCCATTT 3'	[6]
P53	5' TCAACAAGATGTTTTGCCAACTG 3'	5'ATGTGCTGTGACTGCTTGTAGATG 3'	[6]
MCL1	5'-GCCAAGGACACAAAGCCAAT-3	5'-AACTCCACAAACCCATCCCA-3	[7]

References

1. Yu, J.H.; Zheng, G.B.; Liu, C.Y.; Zhang, L.Y.; Gao, H.M.; Zhang, Y.H.; Dai, C.Y.; Huang, L.; Meng, X.Y,; Zhang, W.Y.; Yu, X.F. Dracorhodin perchlorate induced human breast cancer MCF-7 apoptosis through mitochondrial pathways. *Int J Med Sci.* **2013**, 10, 1149-1156.
2. Baharara, J.; Namvar, F.; Ramezani, T.; Mousavi, M.; Mohamad, R. Silver nanoparticles biosynthesized using *Achillea biebersteinii* flower extract: apoptosis induction in MCF-7 cells via caspase activation and regulation of Bax and Bcl-2 gene expression. *Molecules* **2015**, 20, 2693-2706.
3. Tor, Y.S.; Yazan, L.S.; Foo, J.B.; Wibowo, A.; Ismail, N.; Cheah, Y.K.; Abdullah, R.; Ismail, M.; Ismail, I.S.; Yeap, S.K. Induction of Apoptosis in MCF-7 Cells via Oxidative Stress Generation, Mitochondria-Dependent and Caspase-Independent Pathway by Ethyl Acetate Extract of *Dillenia suffruticosa* and Its Chemical Profile. *PLoS One* **2015**, 10, e0127441.
4. Huang, T.C.; Huang, H.C.; Chang, C.C.; Chang, H.Y.; Ou, C.H.; Hsu, C.H.; Chen, S.T.; Juan, H.F. An apoptosis-related gene network induced by novel compound-cRGD in human breast cancer cells. *FEBS Lett.* **2007**, 581, 3517-3522.
5. Sharifi, S.; Barar, J.; Hejazi, M.S.; Samadi, N. Doxorubicin Changes Bax /Bcl-xL Ratio, Caspase-8 and 9 in Breast Cancer Cells. *Adv Pharm Bull.* 2015, 5, 351-359.
6. Madhusoodhanan, R.; Natarajan, M.; Veeraraghavan, J.; Herman, T.S.; Aravindan, N. NFkappaB activity and transcriptional responses in human breast adenocarcinoma cells after single and fractionated irradiation. *Cancer Biol Ther.* 2009, 8, 765-773.
7. Schacter, J.L.; Henson, E.S.; Gibson, S.B. Estrogen Regulation of Anti-Apoptotic Bcl-2 Family Member Mcl-1 Expression in Breast Cancer Cells. *Plos One* **2014**, 9, e100364.
